# The plant growth, water and electricity consumption, and nutrients uptake are influenced by different light spectra and nutrition of lettuce

**DOI:** 10.1038/s41598-023-48284-1

**Published:** 2023-11-25

**Authors:** Hamid Reza Soufi, Hamid Reza Roosta, Mohsen Hamidpour

**Affiliations:** 1 Department of Horticultural Sciences, Faculty of Agriculture, Vali-E-Asr University of Rafsanjan, Rafsanjan, Iran; 2 Department of Horticultural Sciences, Faculty of Agriculture and Natural Resources, Arak University, Arak, Iran; 3 Department of Soil Science and Engineering, Faculty of Agriculture, Vali-E-Asr University of Rafsanjan, Rafsanjan, Iran

**Keywords:** Light responses, Plant sciences

## Abstract

The aim of this study was to investigate the effect of different replacement methods of nutrient solution (complete replacement, electrical conductivity (EC)- based replacement, and replacing based on the plant needs) and different LED light spectra (monochromic white, red, blue, and a combination of red/blue) on the uptake of mineral nutrients, water and electricity consumption and biomass production of two varieties of lettuce (Lollo Rossa and Lollo Bionda; *Lactuca sativa* var. crispa) in the hydroponic systems. The results showed that replacement methods based on the plant needs and based on EC increased shoot fresh mass and yield index in the NFT system. Also, results showed that the combination of red/blue light increased shoot fresh mass and yield index in the NFT system and in the plant factory under treatment by replacement method based on plant needs. Increasing the concentrations of N, K, and Zn and loss of Fe in nutrient solution were observed in all three replacement methods of nutrient solution in the NFT system. Water consumption was decreased under plant nutrition based on plant needs and based on EC. In the plant factory, the application of LED light spectrum also decreased electricity consumption and cost against fluorescent lamps. In general, it is concluded that nutrient solution replacement based on the plant needs and based on EC and the use of different LED light spectra (especially the combination of red and blue light) can be used to reduce the consumption of water and nutrients in the hydroponic cultivation of lettuce.

## Introduction

Global warming can be one of the factors causing extensive and irreparable changes in the production and export of agricultural products in most regions of the world^1^, especially in West Asian countries such as Iran, which are hot and dry regions with little rain. Also migration from villages and production centers to cities have caused two-thirds of energy consumption and more than 70% of carbon emissions^2^. Two billion people of the world are suffering from water shortage and this will reach three billion people by 2050. Indiscriminate extraction of underground water by farmers and industries and wasting water in agriculture by using wrong and high-consumption irrigation methods have caused problems of water shortage, drought and erosion in some regions of the world. Therefore, due to the limitation of Fresh water resources in the world, water management is a vital issue for the global community^3^.

In the traditional methods of cultivation, a large amount of water was used to grow plants because most of them were removed from the reach of the plants and transferred to the cultivation bed, but today, using hydroponic systems has caused a significant reduction in water consumption during the growth period of the plants. The advantages of hydroponics over conventional cultivation include zero-soil cultivation, land-use efficiency, planting environment cleanliness, fertilizer and resource saving, and water consumption reduction^4^. Closed hydroponic systems are one of the hydroponic methods that have more advantages for growing plans such as less consumption of water, and nutrient elements^5^. Also, Recycled hydroponics is practiced to make cultivation cost-effective, sustainable, and environmentally friendly^6^. Another advantage of recycling is that this method of hydroponic makes it possible to convert low-quality water (wastewater) into high-quality water (freshwater)^7^. Researchers indicated that closed systems had more effect on water use efficiency and tomato plant production compared to open systems^5^. However, the uptake of nutrients by plants causes nutritional imbalances in hydroponic recirculating systems^8^. Reuse of nutrient solution in hydroponic cultivation conditions is also one of the ways to reduce water and nutrients and reduce damage to the environment. The recycling of water and nutrients in hydroponic systems is ideal because the drainage can be easily captured for reuse^9^. Cultivation of plants in hydroponic systems as well as NFT and Floating systems can significantly reduce the pollution of water resources, while these methods of cultivation can reduce water and nutrient element consumption^10^. Also, the hydroponic water reuse systems had the most positive overall impacts on small-scale food production systems in the tomato plant^9^. It is reported that the use of the NFT hydroponic system can increase the biomass production of lettuce plants by 6–10% compared to that in the floating raft system and DFT system^11^. Many methods for the reuse of nutrient solutions in different hydroponic systems and in different plants have been examined. It is reported that the recycling of the drainage water resulted in a 33% reduction in potable water used for irrigation in cucumber production and the drainage water contained 59% applied N, 25% applied P and 55% applied K and illustrated the potential for nutrient recovery and production cost savings through the reuse of drainage water^12^. The researchers designed two methods to replace the nutrient solution to store water and nutrients: in the first method nutrient solution was replenished with water and nutrients based on predetermined “uptake concentrations” (UCs), i.e., nutrients to water uptake ratios, following standard commercial practices and in second methods nutrient solution was replenished by applying recalculated UCs of all nutrients after chemical analysis of a drainage solution sample using the software NUTRISENSE (NTS). The results of these researchers showed that uptake concentrations of nutrients determined and the NTS strategy can be successfully used in modern decision support systems (DSS) to optimize nutrient supply and prolong recirculation, thereby saving precious water and nutrients^13^. Researchers found that the use of nutrient solution with high EC could be the reason for the reduction in biomass production, K content in plants, and water consumption of the crop and, increased contents of P, Na, and Cl in the watercress (*Nasturtium officinale*) plants^14^. Thus, optimizing nutrient solutions with new methods, including the replacement of nutrient solution, can change these characteristics. Other studies indicated that recycling nutrient solution reduced biomass production of lettuce and concentration of N, P, K, and Fe and increased Na and Cu levels in the tissue of lettuce, at the same time the negative effects of recycling of nutrient solution were not observed when the recycling solution was either discarded after 2 weeks of use or made using reverse osmosis water and continuously used^15^. It is reported that recycling water can decrease the uptake of phosphate, potassium, and ammonium but the concentration of nitrate and sulfate increased in the drainage solution^16^.

Light quality plays a significant role in the plant growth and uptake of nutrients in lettuce^17^. It is reported that single or combined spectra corresponding to the absorption peaks of photosynthetic pigments such as chlorophyll a and b can increase the ability of plant roots to uptake nutrients^18^.The highest uptake of Ca, Mg, Na, Fe, Mn, Zn, and B has been found in lettuce under the combination of 80% red and 20% blue light^17^. Scientifics reported that the use of continues light treatment significantly increased the concentration of N, P, Fe, and Zn of lettuce in hydroponic system due to the interaction between LED light quality and nutrient liquid nitrogen forms^19^. Liu et al^19^. indicated that treatment of plants by continues red blue LED light can increase the concentration of C, N, P, Ca, and Mg, but this condition had no effect on the concentration of micronutrients. Dengi et al^20^. found that red light increased the concentration of Ca, P, and Mn, but the use of blue light increased N content in okra plant (*Abelmoschus esculentus* L.). Researchers reported that combination of red/blue LED light increased plant growth and nutreint uptake of marigold plant^21^. Also, Vaštakaitė-Kairienė et al^22^. indicated that treatment of spinach with red and blue LED light led to increased growth parameters, mineral nutrients and nutritional quality. It is reported that application of blue LED light had a positive effect on the accumulation of mostly macro- and micronutrients in mustard (*Brassica juncea* ‘Red Lace’) and kale (*Brassica napus* ‘Red Russian’)^23^.

Electricity consumption is also one of the factors that reduce the profit from production in the agricultural sector, especially in greenhouses and plant factories, on the other hand, excessive consumption of electricity can also be a factor in increasing the temperature in the region because power plants produce a lot of heat. The United Nations^24^ forecasts that the countries with the highest consumption of electricity per person are Iceland, Norway, Bahrain, Qatar, Finland, Canada, Kuwait, Sweden, the United Arab Emirates, and the United States of America. Also, the low price of electricity consumption (per kilowatt-hour) in countries like Lebanon, Iran, Ethiopia, Kyrgyzstan, Iraq, and Syria is a factor in increasing electricity consumption in household, industrial, and greenhouse uses, which will cause global warming^24^. LED lamps can be used to reduce electricity consumption in greenhouse conditions because these light sources have a long life and low heat generation compared to high-pressure sodium lamps (HPS). Researchers showed that the use of LED irradiation had a significant effect on biomass production and energy use efficiency of lettuce plants in the NFT and aquaponic systems^25^. The results of studies suggested that the addition of LEDs can effectively remove/degrade/mitigate auto-toxicity in strawberries grown under recycling hydroponics^6^. Thus, the main objective of experiment was to investigate the effect of different replacement methods of nutrient solution (complete replacement, electrical conductivity (EC)- based replacement, and replacing based on the plant needs) and different LED light spectra (monochromic white, red, blue, and a combination of red/blue) on the uptake of mineral nutrients, water uptake, electricity consumption and biomass production of two varieties of lettuce (Lollo Rossa and Lollo Bionda) in the NFT and floating cultivation systems.

## Materials and methods

### Plant material and growth conditions

Two experiments were conducted in a greenhouse under nutrient solution thin layer technique (NFT) and floating systems at the Vali-e-Asr University of Rafsanjan with temperature of 21/15 (day/night), photoperiod of 16/8 hours (day/night), and relative humidity of 50 ± 10% . Seeds of two lettuce varieties (Lollo Rossa and Lollo Bionda (*Lactuca sativa* var. crispa) obtained from Sepahan Rooyesh Isfahan (Isfahan, Iran) and Rijk Zwaan Co (Rijk Zwaan Co., Burgemeester Crezéelaan 40, 2678 KX De Lier, The Netherlands) were sown in seed trays containing perlite substrate and irrigated with distilled water and Resh solution. In the first experiment, twenty days after germination (at four-leaf stage), the seedlings were transferred into small perforated plastic pots containing perlite. These pots were placed in the holes of the floating system. The Resh nutrient solution which formulated for lettuce^26^ (5 mM KNO_3_, 5 mM Ca(NO_3_)_2_, 2 mM MgSO_4_, 1 mM KH_2_PO_4_, 7 μM MnCl_2_, 0.7 μM ZnSO_4_, 0.8 μM CuSO_4_, 0.8 μM Na_2_MoO_4_, 25 μM Fe-EDDHA, and 2 μM H_3_BO_3_, EC: 2.2 dS m^−1^, pH: 6.7) was used (Table 1).

**Table 1:** The concentration of Resh nutrient solution (Resh, 2022) was used in this experiment.

Macronutrients	Concentration (mg L^−1^)	Micronutrients	Concentration (mg L^−1^)
N	139	Fe	0.94
P	31	Mn	0.14
K	215	Zn	0.13
Ca	84	B	0.16
Mg	24	Cu	0.03
S	35	Mo	0.03

**Figure 1:**
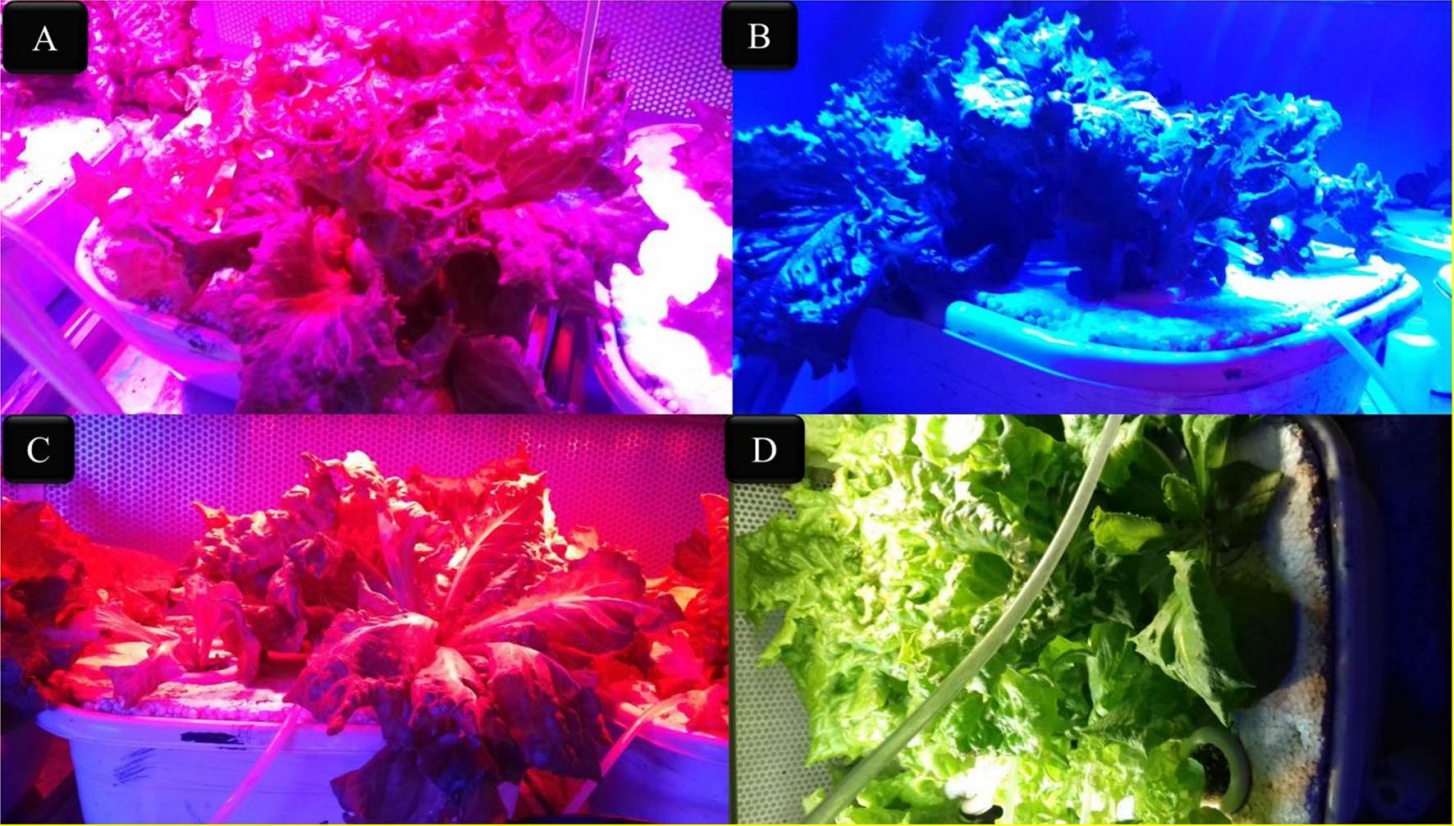
Growing lettuce varieties under different LED light spectra (**A**) red/blue, 3:1; R: B, with a peak 656 nm, (**B**) blue, with a peak at 450 nm, (**C**) red, with a peak at 656 nm, (**D**) white, with a peak at 449 nm, in the floating hydroponic system. The photosynthetic photon flux density (PPFD) was 215 ± 5 μmol m^−2^ s^−1^. The photoperiod of 16/8 h (day/night) was maintained.

The floating system consisted of 24 plastic containers with dimensions of 25 × 30 × 30 cm, which float on a styrofoam. Four plants were cultivated in each container (Fig 1). All culture containers were connected to the air pump (HAILA, model: ACO-388 D) through holes, and the nutrient solution was aerated continuously. After transplanting, seedlings (Four plants in each plastic container) were fed with the Resh nutrient solution. The used nutrient solutions were replaced by three different methods: complete replacement, partial replacement based on EC, and partial replacement according to plant needs.

In complete nutrient solution replacement treatment, the nutrient solution was replaced weekly. For partial replacement according to EC, the EC of the nutrient solution was adjusted to 2.2 dSm^−1^ by adding predetermined amounts of potassium sulfate, calcium nitrate, magnesium sulfate, potassium dihydrogen phosphate, and half strength of micronutrient solution every 48 h. In the replacement treatment based on the needs of the plants, potassium nitrate is used at the same concentration as in the original solution, and the amount of calcium nitrate, magnesium sulfate, and potassium dihydrogen phosphate was reduced by three quarters and the microelements (Fe, Zn, Cu and Mn) were reduced by half and added every two days based on the amount of water added to the plastic container in which the plants were cultivated

**Figure 2:**
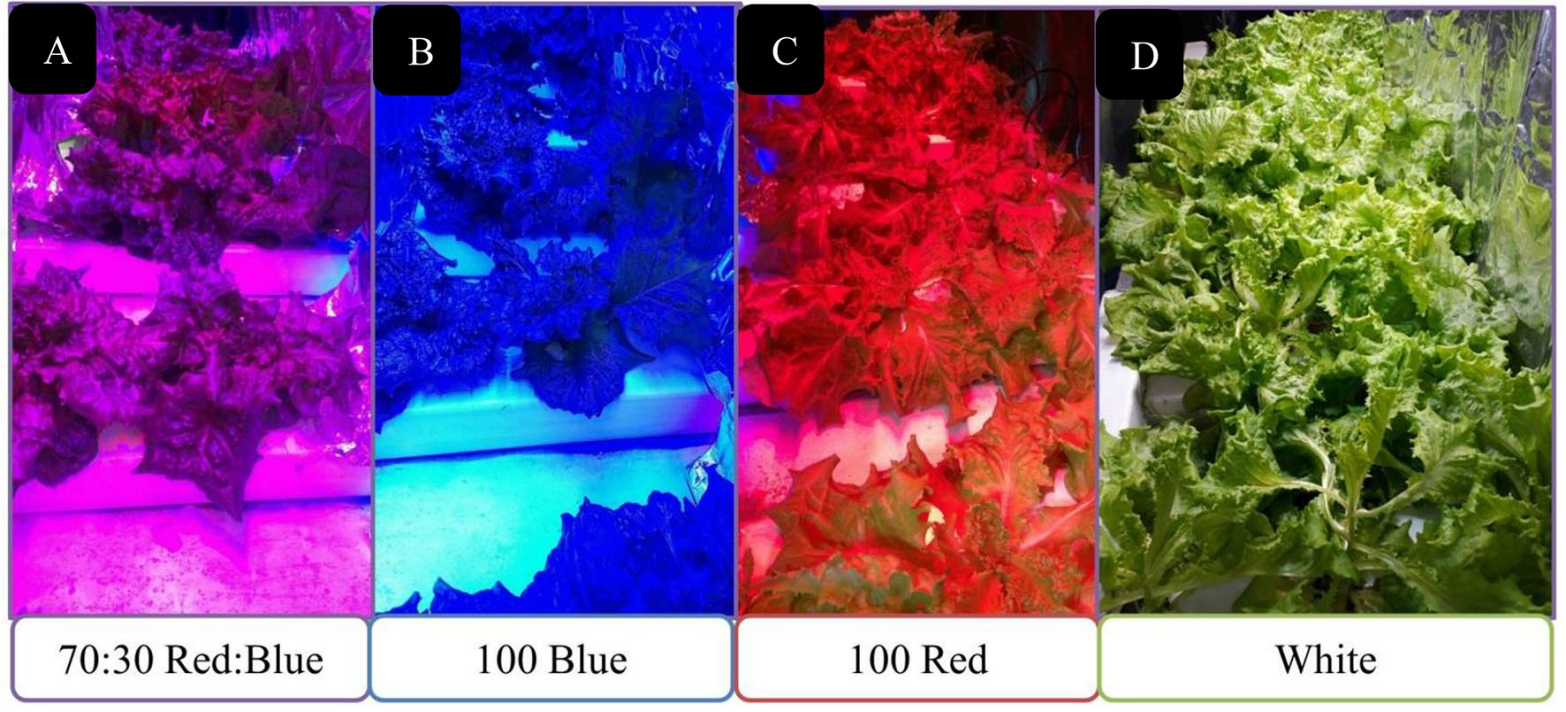
Growing lettuce varieties under different LED light spectra (**A**) red/blue, 3:1; R: B, with a peak 656 nm, (**B**) blue, with a peak at 450 nm, (**C**) red, with a peak at 656 nm, (**D**) white, with a peak at 449 nm, in the NFT hydroponic system. The photosynthetic photon flux density (PPFD) was 215 ± 5 μmol m^−2^ s^−1^. The photoperiod of 16/8 h (day/night) was maintained.

In the second experiment, plants were grown in an NFT system located in the experimental greenhouse (Fig 2). Seeds of lettuce were sown in the seed trays filled with fine perlite. After the appearance of the forth leaf, the seedlings were transplanted to the small plastic net pots containing horticultural grade perlite medium. These small pots were placed in growth holes in the NFT channels (dimensions: 200 cm × 20 cm × 12 cm). Two NFT channels (one unit) were connected to a reservoir (50 L). Each of the two channels was connected to a vertical gully for efficient transfer of nutrient solution to the reservoir. On each channel, there were 12 holes with a distance of 20 cm from each other. A submersible water pump was used to circulate nutrient solutions in the NFT system.

To allow uniform flow of nutrient solution, gully inclination was 1%. The flow rate on the outlet of the NFT system was 2.4 L min^−1^.After transferring the plants to the NFT systems, the lettuce seedlings were fed with nutrient solutions similar to those described for the first experiment. The EC and pH were monitored daily during the cultivation process using portable pH meter (Fisherbrand™ accumet™ AP115 Portable pH Meter Kit) and portable EC meter (HI9033 EC Meter, Setare Arsh Aria company).

### LED tubes and the light treatments

In this study, lettuce cultivars were treated by different LED lamps with 24 × 3W with 90% efficiency, 40 cm × 10 cm light coverage area, 600 mA ± 5% output current, and 50/60 Hz output frequency (Parto Roshd Novin Company, Iran) with different spectral ranges: white (W, with a peak at 449 nm), blue (B, with a peak at 450 nm), red (R, with a peak at 656 nm) and red/blue (3:1; R: B, with a peak 656 nm). The photosynthetic photon flux density (PPFD) was 215 ± 5 μmol m^−2^ s^−1^ in all treatments. The photoperiod of 16/8 h (day/night) was maintained. The LED light systems were placed 30 cm above each plant (Figs. 1 and 2).

### Vegetative characteristics

At the end of the experiment (42 days after transplanting), the plants were harvested, and shoot fresh mass (SFM) was measured with a digital scale with an accuracy of 0.001 g. According to the number of plants per unit area and the total weight of plants in each treatment, weight yield (head weight) per unit area was determined and finally, the yield index was calculated based on plant weight per square meter.

### Mineral nutrient analysis

The dry weights of shoots were recorded, and the shoots were ground using a stainless-steel grinder. The total concentrations of N, P, K, Ca, Mg, S, Fe, Mn, Zn, Mo, B, Na and Cl in plants shoots were determined according to standard methods following H_2_SO_4_ digestion (Estefan et al., 2013).

### Uptake and loss

Two methods were used to determine the uptake of nutrients by plant^27^. Normally, the increase in the nutrient content of the product in the crop is equal to the loss of nutrients from the nutrient solution. The uptake by the crop is defined as:

$${\text{U}} = \, \left( {{\text{A}}_{{\text{t}}} - {\text{A}}_{{{\text{t}} - {1}}} } \right) \, \times {\text{ N}}$$

where U (mmol week^−1^) is the total uptake by N plants during the time interval of one week. A_t_ and A_t-1_ (mmol) are the nutrient contents of a plant at the end and the start of the week, respectively. The loss from nutrient solution is defined as:

$${\text{L}} = \, \left( {{\text{C}}_{{{\text{t}} - {1}}} - {\text{C}}_{{\text{t}}} + {\text{ D}}} \right) \, \times {\text{ V}} - {\text{S}},$$

where L (mmol week^−1^) is the loss of nutrients from the solution, Ct and Ct − 1 (mmol L^−1^) are the nutrient concentrations at the end and start of a week, respectively and D (mmol) is the total amount of nutrient added to the supply tank, V (L) is the constant volume of the supply tank, and S (mmol week^−1^) represents the removal of nutrient due to frequent sampling of the nutrient solution.

### Experimental design and data analysis

The experiment was designed as a 2×3×4 factorial and arranged in a completely randomized design with three replications. Also, SAS software version 9.4 (SA Institute, Cary, NC, USA) was used to analyze the data. A two-way analysis of variance (ANOVA) was used for statistical analysis of data. In addition, Duncan's multiple range test was used at the 5% probability level when the analysis of variance showed significant treatment effects.

### Ethical approval

The authors confirm that all the experimental research and greenhouse studies on lettuce plants, including the collection of plant material, complied with relevant institutional, national, and international guidelines and legislation.

## Results

### Plant growth

**Figure 3:**
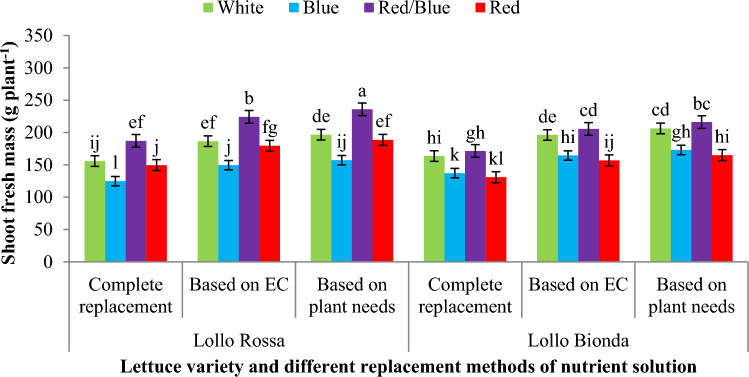
The effect of different replacement methods of nutrient solution (complete replacement method, replacement method based on EC and replacement method based on plant needs) and different light spectra (red/blue, 3:1; R: B, with a peak 656 nm, blue, with a peak at 450 nm, red, with a peak at 656 nm, white, with a peak at 449 nm), on the shoot fresh mass of lettuce varieties in the NFT system. The photosynthetic photon flux density (PPFD) was 215 ± 5 μmol m^−2^ s^−1^. The photoperiod of 16/8 h (day/night) was maintained.

**Figure 4:**
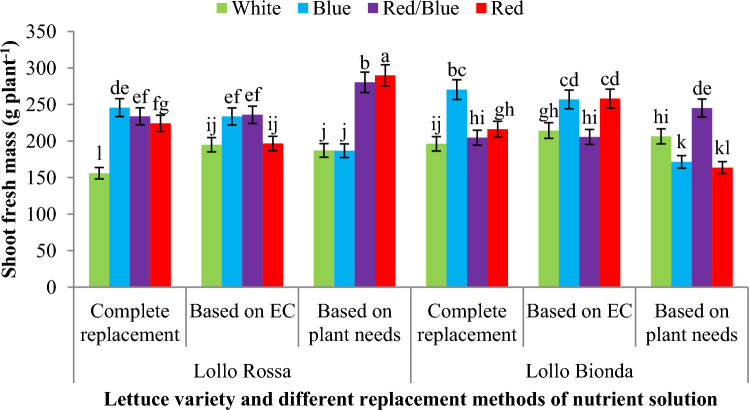
The effect of different replacement methods of nutrient solution (complete replacement method, replacement method based on EC and replacement method based on plant needs) and different light spectra (red/blue, 3:1; R: B, with a peak 656 nm, blue, with a peak at 450 nm, red, with a peak at 656 nm, white, with a peak at 449 nm), on the shoot fresh mass of lettuce varieties in the floating system. The photosynthetic photon flux density (PPFD) was 215 ± 5 μmol m^−2^ s^−1^. The photoperiod of 16/8 h (day/night) was maintained.

The shoot fresh mass and yield index were significantly influenced by the applied treatments in NFT and floating systems. Results also showed that in NFT system, nutrition based on plant needs and nutrient solution EC and combination of red and blue LED light increased the shoot fresh mass in Lollo Rossa lettuce variety compared to control treatment (white LED and complete replacement) but monochromic red and blue LED light could not improve the shoot fresh mass of Lollo Rossa variety compared to the control treatment. Also our results showed that the treatment of Lollo Bionda variety with the combination of red and blue light caused an increase in the shoot fresh mass compared to other light spectrums in all three replacement methods of nutrient solution (Fig. 3). Results also showed that the monochromic red LED spectrum and combination of red/blue LED light had more effect on shoot fresh mass compared to the white LED spectrum in the Lollo Rossa variety that fed based on plant needs, however in this lettuce variety, the combination of red/blue and monochromic blue under nutrition based on EC and monochromic blue, red/blue and monochromic red LED light under complete replacement also increased the shoot fresh mass against to control (white LED spectrum and complete replacement) in floating hydroponic (Fig. 4). In the Lollo Bionda lettuce variety, monochromic blue LED light under complete replacement and monochromic blue and red/blue LED spectra under fed based on EC and monochromic red and combination of red/blue under nutrition based on plant needs had the highest shoot fresh mass against to control (white LED spectrum and complete replacement) in floating hydroponic (Fig. 4).

### Yield index

**Figure 5:**
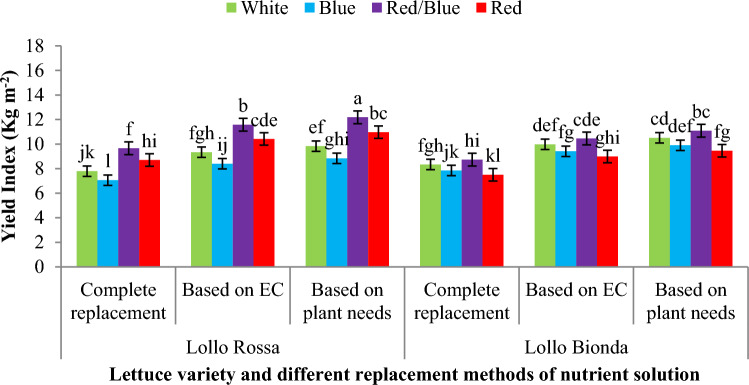
The effect of different replacement methods of nutrient solution (complete replacement method, replacement method based on EC and replacement method based on plant needs) and different light spectra (red/blue, 3:1; R: B, with a peak 656 nm, blue, with a peak at 450 nm, red, with a peak at 656 nm, white, with a peak at 449 nm), on the yield index of lettuce varieties in the NFT system. The photosynthetic photon flux density (PPFD) was 215 ± 5 μmol m^−2^ s^−1^. The photoperiod of 16/8 h (day/night) was maintained.

**Figure 6:**
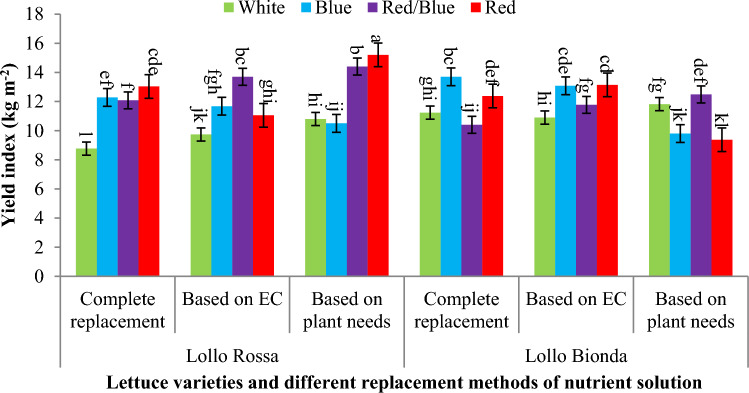
The effect of different replacement methods of nutrient solution (complete replacement method, replacement method based on EC and replacement method based on plant needs) and different light spectra (red/blue, 3:1; R: B, with a peak 656 nm, blue, with a peak at 450 nm, red, with a peak at 656 nm, white, with a peak at 449 nm), on the yield index of lettuce varieties in the floating system. The photosynthetic photon flux density (PPFD) was 215 ± 5 μmol m^−2^ s^−1^. The photoperiod of 16/8 h (day/night) was maintained.

Results showed that the combination of reb/blue and monochromic red LED light had more effect on yield index in Lollo Rossa lettuce variety in all three replacement methods of nutrient solution compared to control treatment (white LED light and complete replacement), while the monochromic decreased yield index compared to white LED light all three replacement methods of nutrient solution (Fig. 5). Also, the results showed that in Lollo bionda variety, the combination of red and blue light in all three methods of replacement of nutrient solution caused an increase in yield index compared to monochromatic red and blue light, but there was no statistically significant difference compared to white LED light (Fig. 5). Results also showed that monochromic red LED spectrum and combination of red/blue LED light caused the highest amount of yield index against to white LED spectrum in the Lollo Rossa plant that fed based on plant needs, although in this lettuce variety, combination of red/blue and monochromic blue and red LED spectra under nutrition based on EC and complete replacement also improved the yield index compared to the control treatment (white LED spectrum and compete replacement) in floating hydroponics (Fig. 6). In Lollo Bionda lettuce variety, monochromic blue and red LED light under complete replacement and fed based on EC and combination red/blue in nutrition based on plant needs had the highest yield index against to control (white LED spectrum and compete replacement) in floating hydroponics (Fig. 6).

### EC changes and water consumption in NFT system

**Figure 7:**
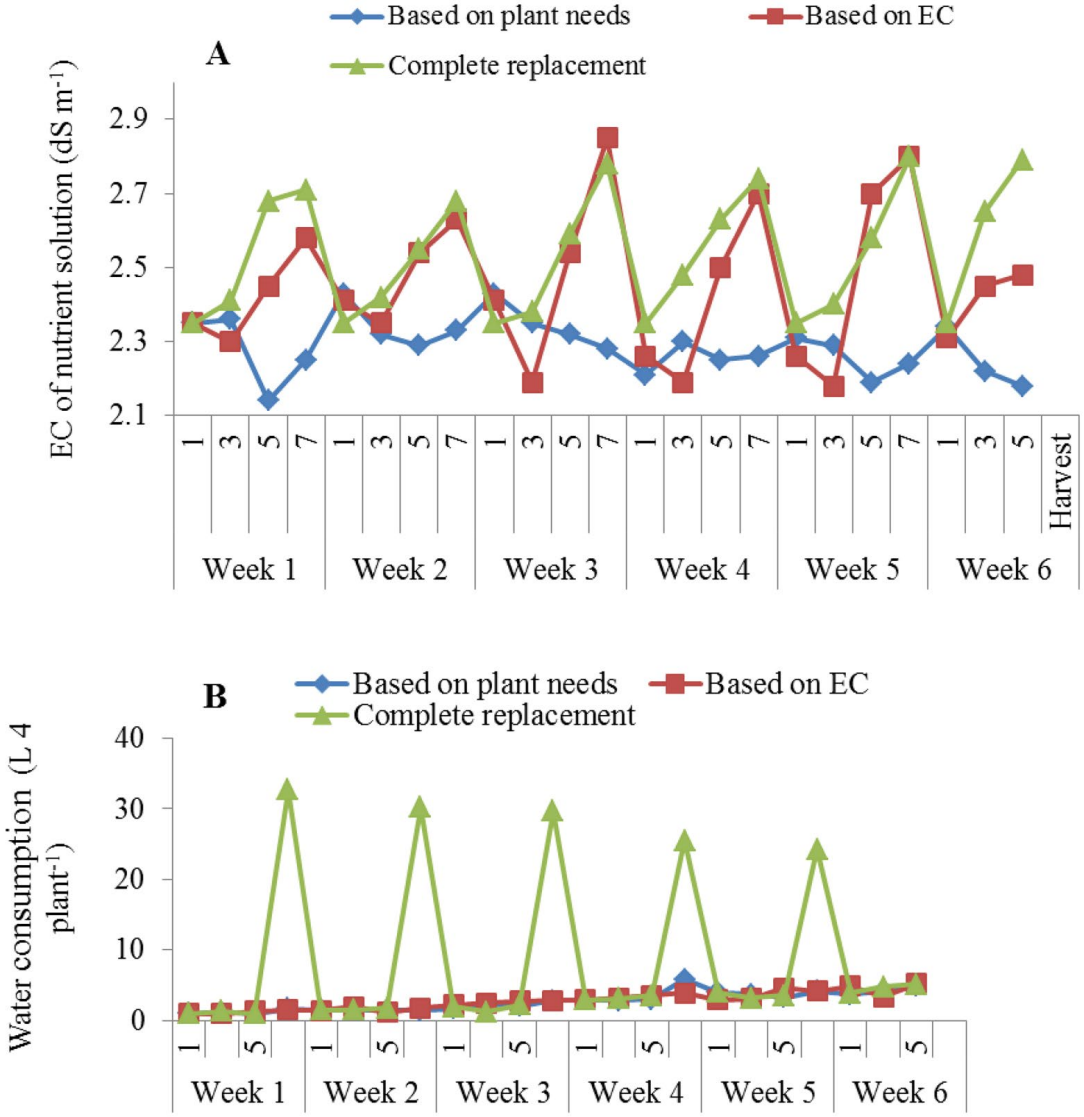
(**A**) EC of nutrient solution and (**B**) Water consumption during the 42-day growing period of lettuce varieties in the NFT System.

**Figure 8:**
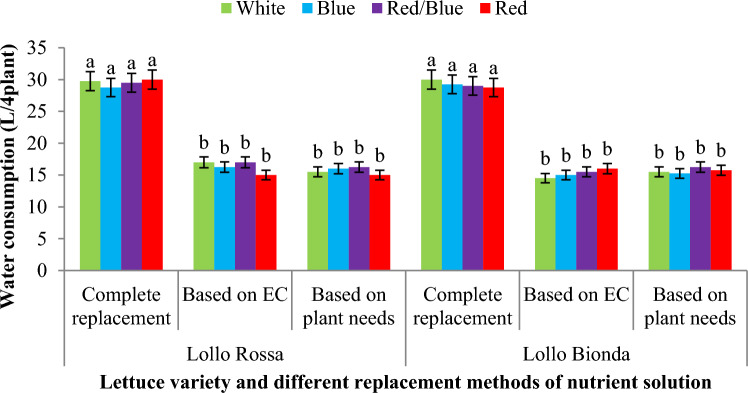
The effect of different replacement methods of nutrient solution (complete replacement method, replacement method based on EC and replacement method based on plant needs) and different light spectra (red/blue, 3:1; R: B, with a peak 656 nm, blue, with a peak at 450 nm, red, with a peak at 656 nm, white, with a peak at 449 nm), on the water consumption of lettuce varieties in the NFT system. The photosynthetic photon flux density (PPFD) was 215 ± 5 μmol m − 2 s − 1. The photoperiod of 16/8 h (day/night) was maintained.

The results showed that replacement methods based on EC and complete replacement of nutrient solution recorded the highest changes in EC during the growth period of lettuce varieties in NFT system under treatment with different LED spectra (Fig. 7A). The lowest fluctuation was observed in the nutrient solution that replenished based on the plant needs (Fig. 7A). Also, the highest water consumption was recorded in the conditions of complete replacement of the nutrient solution compared to another replacement method of nutrient solution (Fig. 7B). At the beginning of the growing period of lettuce varieties, due to the low absorption of water and mineral nutrients by plants, the amount of waste water nutrient solution was high in the complete replacement method of nutrient solution, but in over time, with the increase in plant growth and greater demand for water and nutrients, the waste water and nutrient solution was reduced in NFT system (Fig 7B). During 42 days of lettuce growth, for each LED light spectrum, an average of 60, 15, and 15 liters of water were used for plants fed based on complete replacement of nutrient solution, replacement based on EC, and replacement based on the plant needs, respectively. It was also found that the complete replacement of the nutrient solution after one week caused a significant increase in water consumption during the growth period of lettuce (42 days) in the NFT system compared to the other treatments (Fig. 8).

### Mineral nutrient uptake

**Figure 9:**
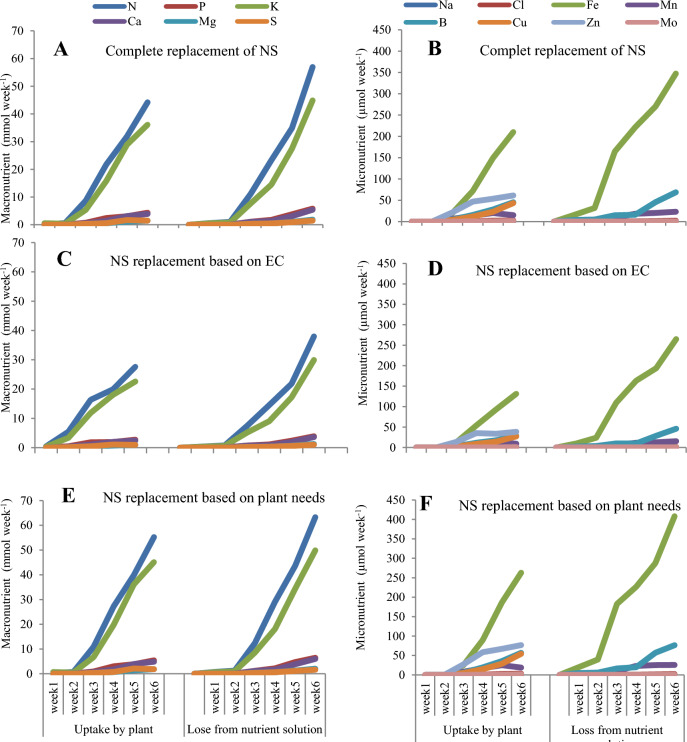
Mineral nutrients uptake by plant and loss from nutrient solution during the 42-day growing period of lettuce varieties treated with different replacement methods of nutrient solution (**A**, **B**) Complete replacement method, (**C**, **D**) Replacement method based on (**E**, **C**) and (**E**, **F**) Replacement method based on plant needs) in NFT system.

The results of the present study showed that in all replacement methods of nutrient solution, nitrogen and potassium were absorbed in high levels by the plants compared to other nutrients and consequently their concentration decreased in the nutrient solution (Fig. 9A,C,E). So that after two weeks of the growing period of lettuce plants, this elevation in the absorption of nutrient elements was seen and showed an increasing trend until the end of the growing period. Also, under these conditions, iron was absorbed by plants higher than other micro-nutrients, but a high amount of iron was lost from the nutrient solution (Fig. 9B,D,F). It was also found that the absorption of N, K, and Fe by plant and loss of N, K, and Fe from nutrient solution in the replacement methods based on plant needs was more than the replacement based on EC and complete replacement method of nutrient solution (Fig. 9A–F).

### EC changes and water consumption under floating system

**Figure 10:**
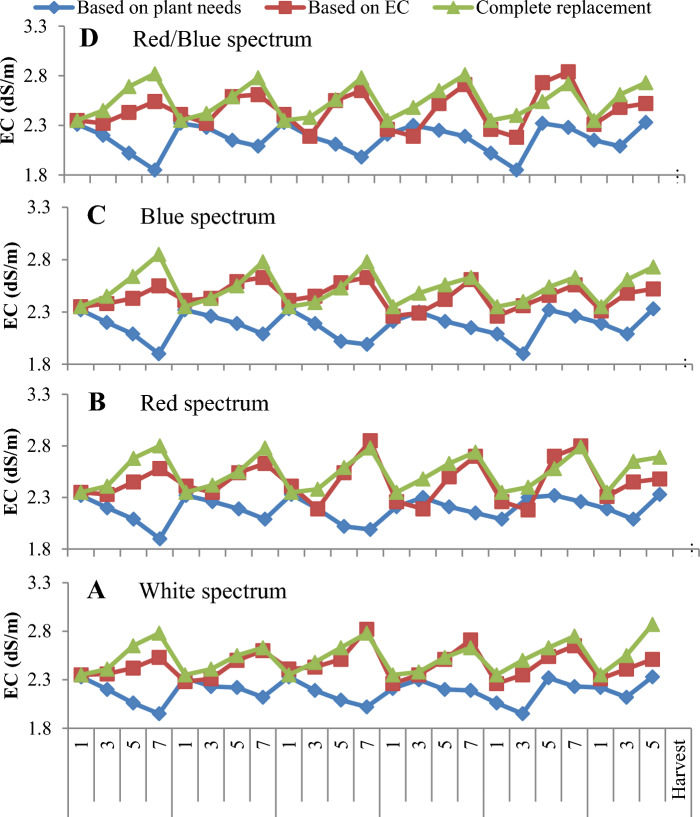
EC changes during the 42-day growing period of lettuce varieties in the floating system with different replacement methods of nutrient solution (complete replacement method, replacement method based on EC and replacement method based on plant needs) and different LED spectra ((**A**): White spectrum, (**B**): Red spectrum, (**C**): Blue spectrum: (**D**), Red/blue spectrum).

**Figure 11:**
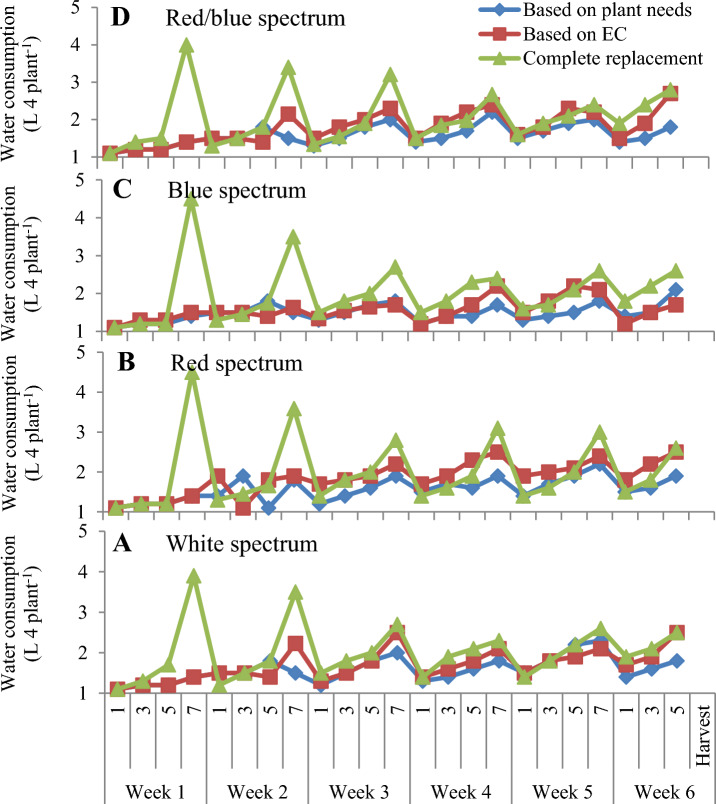
Water consumption during the 42-day growing period of lettuce varieties in floating hydroponic system with different replacement methods of nutrient solution (complete replacement method, eeplacement method based on EC and replacement method based on plant needs) and different LED spectra ((**A**): White spectrum, (**B**): Red spectrum, (**C**): Blue spectrum: (**D**), Red/blue spectrum).

The results showed that the replacement method of nutrient solution based on EC and complete replacement caused the most changes in the EC of the nutrient solution compared to the plants that fed based on the plant needs under treatment with different LED light spectra (Fig. 10A–D). In addition, the most EC changes were observed in plants treated with monochromatic red and combination of red and blue LED light in floating systems (Fig. 10B,D). The use of complete replacement of nutrient solution and replacement based on EC caused an increase in the amount of water consumption compared to the plant that fed based on the plant needs under the all spectrums of LED light in floating culture conditions (Fig. 11A–D). In addition, it was found that the highest changes in water consumption were in plants that treated with white light and combination of red and blue LED light (Fig. 11A,D). At the beginning of the growth period of lettuce varieties, due to the low absorption of water and mineral nutrients by plants, the amount of wastewater nutrient solution was high in the complete replacement method of nutrient solution, but over time, with the increase in plant growth and greater demand for water and nutrients under all LED light treatment, the wastewater and nutrient solution was reduced in the floating system (Fig. 11A–D).

### Electricity consumption and electricity cost

**Figure 12:**
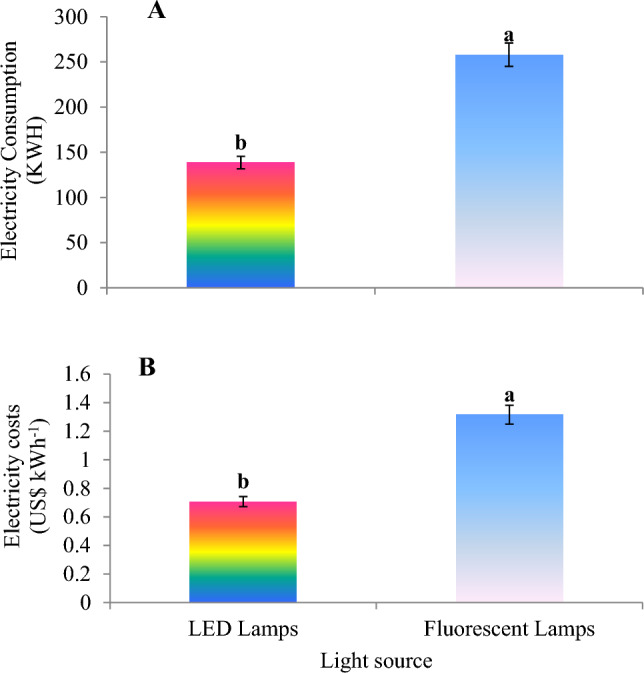
Electricity consumption (**A**) and electricity cost (**B**) during the 42-day growing period of lettuce in a floating hydroponic system treated by LED and Fluorescent lamps. The photosynthetic photon flux density (PPFD) was 215 ± 5 μmol m^−2^ s^−1^. The photoperiod of 16/8 h (day/night) was maintained.

The results of our investigation showed that the treatment of LED light on lettuce varieties in the floating system in the plant factory caused a 46.2% decrease in electricity consumption compared to the fluorescent light source (Fig 12A). Also, the highest cost of electricity consumption was caused by the light treatment of fluorescent lamps on lettuce varieties, but the use of LED lights caused a 47.7% reduction in the cost of electricity consumption in this study (Fig 12B). Of course, due to the low cost of electricity consumption in Iran, there is still a tendency to use fluorescent and high-pressure sodium lamps in the greenhouse and plant factory conditions, but with the modification of cultivation methods and the use of vertical cultivation and LED light can significantly reduce electricity consumption and costs and play an effective role in the sustainability of the environment.

## Discussion

### Plant growth traits

Based on the results of our investigation, all three replacement methods of nutrient solution and combination of red/blue LED light increased the shoot fresh mass and yield index of lettuce varieties in the NFT system and floating hydroponic. In the floating system, the Lollo Rossa lettuce variety that fed based on plant needs and a combination of red/blue and monochromic red LED light showed the highest shoot fresh mass and yield index compared to the control treatment (complete replacement and white LED light), but in Lollo Bionda, use of monochromic blue LED light had the more effect on shoot fresh mass and yield index in complete replacement and replacement method based on EC against to control treatment (complete replacement and white LED light). The increase in the mentioned characteristics can be due to the optimization of the conditions of the nutrient solution in terms of electrical conductivity, which has a significant effect on the absorption of macro and micro-nutrients, so that the fluctuation of the nutrient solution electrical conductivity can cause the decrease of nutrients uptake and reducing the transfer of nutrients from the roots to the shoots. The increase in the electrical conductivity of the nutrient solution caused by the accumulation of macro-nutrients such as sulfates and phosphates and the creation of salinity stress by affecting the osmotic pressure and water potential is an obstacle in the direction of the transfer of water and nutrients to the roots. Jagtap et al^28^. with study on three nutrient compositions for lettuce cultivars in two hydroponic systems indicated that the use of proper EC can increase the vegetative growth in lettuce cultivar in NFT system. In our study results showed that the use of replacement method based on plant needs with the proper EC for lettuce cultivar was more suitable for plant growth. In other study, has been reported that the lettuce cultivars in the saline condition had a lower yield^29^ that agreed with our results in the high EC in replacement methods based on EC and complete replacement with negative effect of yield index. The changes in the nutrient formulation required by plants has positive effects on the growth and yields characteristics of lettuce plants, as in this research, the concentration of some macro and micro- elements was changed in the nutrient solution based on the plant needs, and it caused an increase in the growth and yield characteristics of lettuce varieties; which is in accordance with the previous study results obtained on lettuce in raft and floating hydroponic systems^30, 31^. Researchers revealed that the use of species-specific replacement of nutrient solution formulated for arugula and basil plants had the lowest effect on plant growth and nutrient uptake by plants when compared to nutrient replenishment with a standard hydroponic solution used commercially^32^. In our study pH of nutrient solution increased above 7 in replacement methods based on EC and complete replacement during the growth periods that can cause Mn^2+^, PO_3_^4–^, Ca^2+^, and Mg^2+^ to precipitate. Resh^26^ reported that in pH above 7, the nutrients availability by plants are restricted. Also excessive amount of nutrient elements in pH above 7 can increase EC of nutrient solution and reduce uptake of other nutrient elements^26^. The high electrical conductivity can also increase the amount of N and K uptake by the leaves^33, 34^ that agreed with our results. Inverse relationship of plant growth and yield with nutrient uptake rate could be due to negative impacts of excessive absorption of nutrient elements on plant growth and yield in the forms of toxicities, imbalance or defensive response that we observed in the methods of complete replacement and replacement based on EC. Plants uptake more K than any other element, with the exception of N^35^. The increase in the EC of the nutrient solution after two weeks caused the toxicity of the remaining nutrients elements in the nutrient solution^33^ that agreed with our results. Excessive absorption of nutrients such as N, K and Fe can cause the imbalance of nutrients and the toxicity of these nutrients in plants, as it has been stated in pervious researches that excessive amount of K in plants causes a decrease in the concentration of P and Mg in pepper plants^36^. In the current study, high uptake of N, K and Fe elements caused an ionic imbalance in the nutrient solution and prevented the absorption of macro and micro-nutrient elements. It is reported that K, Ca, and Mg compete with each other and excessive absorption of each nutrient will reduce the uptake rate of the other two^37^. A study on maize plant indicated that increase levels of K decreased the P, Mg and Ca concentration in both leaves and roots of maize^38^. As stated in the results of this study, the highest concentrations of N, K and Zn was observed in the plants that fed based on plant needs. The increased uptake of these elements was a factor in increasing the growth characteristics of plants in NFT conditions because these elements have a direct role in the growth, development and photosynthesis of plants^39^. Increase in K concentration of arugula plant under high EC and significant decline in Ca and most of the micronutrients including Fe, Mo, Cu, B and Mn has been reported^40^. High consumption of water and nutrients in the conditions of complete replacement of the nutrient solution and extensive changes in the EC of the nutrient solution in the NFT system were observed, which could be related to the remaining high macro-elements in the nutrient solution. An increase in the concentration of elements in the nutrient solution causes an increase in EC and thus prevents the uptake of nutrients by plants and osmotic pressure on the roots of plants in hydroponic cultivation conditions. Nutrient imbalance, such as high Ca, Mg, and S concentration around the root is one of the causes to the reduction of vegetative parameters^41^ that we observed in the complete replacement of nutrient solution. On the other hand, the use of nutrient solution with the proper nutrient elements balance caused the increase of water and nutrient uptake, and accordingly of plant growth^42^. In other studies, researchers indicated that reduction of plant growth can happen under the disturbed mineral balance of nutrient solution^43^. The effect of different nutrient solutions on plant growth was previously reported on lettuce^29, 33, 44, 45^, tomatoes^46^ and artichokes and cardoon^47^, which are consistent with our results. In this research, lettuce varieties under replacement methods based on plant needs and EC had higher shoot fresh mass and yield index compared to complete replacement. In other study results showed that the changes of nutrient solution after two weeks can reduce fresh and dry mass of lettuce plants^33^.

### Mineral nutrient concentration

The results of our study also showed that the amount of uptake of N, K, and Zn elements increased by the varieties in all three replacement methods of the nutrient solution under treatment with different LED light spectra, and a high amount of Fe was lost from the nutrient solution. Nitrogen plays an important role in the photosynthetic apparatus and chlorophyll biosynthesis; also the opening and closing of the stomata and the timely reaction of the stomatal guard cells to gas exchange are dependent on the K element. Furthermore, Zn and Fe elements are among the main elements of the photosynthetic cycle, and a decrease in the uptake of these elements can cause irreparable damage to plants^48^. The decrease in the amount of uptake of other nutrients in all three replacement methods of nutrient solution (except N, K, and Zn) may be due to (i) decreasing water potential, (ii) disruption of ion balance, and (iii) decreasing root growth^49^. Measurement of solution electrical conductivity (EC) is helpful, but if the solution is appropriate, low EC usually means healthy plants and active nutrient uptake^50^. The high consumption of water and more uptake of nutrient elements (especially N, K) and an increase in shoot fresh mass and yield index may be associated with photosynthetic characteristics. Because the increase in water uptake leads to increase in the nutrients uptake by the roots and transfer of the nutrients to the aerial organs, which results in an increase in the production of carbohydrates. Carbohydrates are the main requirement for the growth and development of plant organs, including aerial organs and roots. Excessive EC can reduce nutrient elements uptake, especially Ca^2+^^51, 51^. Researchers found that an increase in EC can reduce water absorption capacity and Ca uptake in tomato plants^52^. It is reported that increasing of the EC of the nutrient solution of tomato plants reduced P concentration, increased K, and did no effect on N concentration^52^. In the present experiment, complete replacement of nutrient solution and replacement method based on EC with high changes in EC of the nutrient solution increased the uptake of K and N and decreased Ca concentration in both lettuce varieties in 42-day growing periods. The species-specific replenishment of nutrient solution can decrease the concentration of nutrient ions (especially Ca, Mg and S) against a standard hydroponic replenishment solution in arugula, and basil plant^32^. Researchers believed that complete replacement of nutrient solution after two weeks could be selected as the best nutrient concentration to be used for growing leaf lettuce in stationary culture^33^; but our results showed that the use of the replacement method based on plant needs can increase the nutrient elements uptake and reduce water and nutrient elements consumption in NFT and floating hydroponic systems during 42 days. The combination of different light spectrums has been shown enhanced the uptake of K, Ca and Mg in comparison to control plants grown under conventional HPS lamps and spectral changes of the red and blue light were significantly influenced the uptake of Fe and Zn^53^, which is consistent with the results of the present study. The results of our investigation showed that the use of different light spectrums in all three methods of nutrient solution replacement caused an increase in the uptake of N, K, Fe and Zn nutrients (Fig. 9).

### Effect of LED spectra on growth and nutrient concentration

It was also found that using the combination of red and blue light in both varieties of lettuce can increase the shoot fresh mass and the yield index of the varieties of lettuce. The role of red light in the response to stomatal conductance is independent of the concurrent photosynthetic rate of the guard cells or of that of the underlying mesophyll^54^ and blue light is the response of chloroplast sub-compartment proteins, including those active in stomatal opening and closing, and leaf physiological and indicated induced growth enhancement^55, 56^, the combination of these two light spectra can increase the production photosynthetic properties and increase the gas exchange that is the main factor in absorbing nutrients and producing primary and secondary metabolites for plants. Marigold plants that were exposed to red, blue, and a combination of red/blue light had the highest growth traits and photosynthetic pigments (chlorophyll a, b, total, and carotenoid concentrations)^57^ which agreed with the results of this study. The changes in EC and water consumption under the combination of red and blue LED light were more than other light spectra used in this study, and this could be due to the increase in the activity of the photosynthetic apparatus, gas exchange through the stomata and accordingly, the increase in the absorption of nutrients by the roots and transfer them to aerial organs. Under these conditions, some of the macro-elements that are least absorbed remain in the nutrient solution and cause changes in the electrical conductivity of the nutrient solution during the growth period of lettuce plants. According to the literature, blue light causes an opening of ion channels located on cell plasma membranes and promotes the flux of ion transport through the control of the blue light receptor phototropin (Phot 1 and Phot 2)^58^. Also, other studies reported that red and blue light had a positive effect on mineral nutrient content in buckwheat and beet microgreens^59–62^. So the combination of red and blue light can be more effective on water and mineral uptake by plants, especially lettuce varieties. On the other hand, manipulation of the light environment to adjust its nutrient absorption capacity can further increase the yield and economic value of leafy crops^58^.

## Conclusion

All growers to avoid nutritional disorders typically, dump and replace the hydroponic solution periodically, which is wasteful, has an economic cost and it also harms the environment. Replenishing the consumed water and nutrient elements with fresh solution and maintaining a constant solution electrical conductivity (EC) is a common nutrient management strategy that can lead to ion accumulation and nutrient imbalances since nutrients are taken up by roots and depleted from solution at different rates. In this study, the use of a replacement method based on plant needs had more effect on fresh mass and yield index, and the combination of red and blue light in the Lollo Rossa lettuce variety fed based on plant needs increased shoot fresh mass and yield index. Water consumption was decreased under plant nutrition based on plant needs and based on EC. In the plant factory, the application of LED light spectrum also decreased electricity consumption and cost against fluorescent lamps. In general, it can be said that feeding varieties of lettuce based on the plant needs and illumination by LED lights has a positive effect on the growth characteristics, yield index, saving water and energy, and absorption of nutrients in the NFT and floating hydroponic systems and it is recommended for growing lettuce varieties in greenhouse and plant factory with closed hydroponic systems.

## Data Availability

The datasets generated during and/or analysed during the current study are available from the corresponding authors on reasonable request.
